# Splenectomy for Congestive Hypertrophy

**Published:** 1900-08

**Authors:** J. Wesley Bovée

**Affiliations:** Washington, D. C., Clinical Professor of Gynecology in Columbian University and Gynecological Surgeon to Columbia, Providence and Columbian University Hospitals.


ATLANTA

### Journal-Record of Medicine.

Successor to Atlanta Medical and Surgical Journal, Established 1855, and Southern Medical Record, Established 1870.

VOL. II. AUGUST, 1900. No. 5.

EDITORS { BERNARD WOLFF, M.D., DUNBAR ROY, A.B., M.D. — M. B. HUTCHINS, M.D., BUSINESS MANAGER.

PUBLISHED MONTHLY. OFFICE 305 AND 306 FITTEN BUILDING.

### *ORIGINAL COMMUNICATIONS.*

### SPLENECTOMY FOR CONGESTIVE HYPERTROPHY.*

BY J. WESLEY BOVÉE, M.D., WASHINGTON, D. C.,

Clinical Professor of Gynecology in Columbian University and Gynecological Surgeon to Columbia, Providence and Columbian University Hospitals.

In the *Medical News*, 1899, Vol. lxxv, p. 848, I reported the history of two cases of splenectomy, with a general résumé of the literature of the subject up to that time. One of those operations was done for a leucocythemic spleen weighing twelve pounds and seven ounces, and, as a complication, she had clubbing of the cardiac valves. Death occurred from shock just as the tumor was being pulled from the abdominal cavity. It seemed to be that the support to the diaphragm and heart from pressure by the large spleen had been gradually increased by the growth of the tumor, and now

* Read by invitation at the Annual Meeting of the Medical Association of Georgia, at Atlanta, April 18, 19, and 20, 1900.

was necessary to prolong life: as soon as it was removed by taking away the tumor collapse immediately occurred. I now believe removal of the enlarged organ under such conditions, even though the heart be normal, is absolutely unjustifiable.

The other case was one of enlarged, movable spleen associated with tubercular peritonitis and splenitis, the organ weighing twenty-eight ounces—about four times its normal weight. In this instance the tumor was successfully removed, and the patient was alive several months later, though she was thought to be suffering from pulmonary tuberculosis. At the time the paper mentioned was prepared I had another enlarged spleen to deal with, and under peculiar circumstances. She was therefore kept under observation nearly three months before the operation. She was in the hospital during the two months just preceding the operation, and opportunities for carefully studying her condition were afforded and accepted. In this case careful and frequent blood counts were made, as well as a complete record of her pulse and temperature, and many other details preceding operation. The changes in her condition subsequent to operation were given in detail, and show how little harm results from extirpation of even such a large spleen as was this one, and, *au contraire,* the signal benefit to be derived from its removal—an improvement that was not possible of attainment in any other manner. Considerable emphasis is placed upon the importance of observation of splenectomy cases, other than emergency operations, for a considerable time previous to operation in order to be certain of the blood conditions, and to attempt to at least arrive at a conclusion as to the actual cause and character of the splenic condition. This may be materially aided by continuing the study after operation. This is absolutely necessary, as it is well understood that not all cases of enlarged spleen are amenable to extirpation. Nearly as many cases of splenectomy have ended fatally as have ended successfully, and for the very reason that the blood conditions had not been studied before operation, the advantage of which was not realized or understood, and as a result the operation has been many times done in cases in which we know to-day it is absoluely contraindicated.

The principal reason usually for removal of the spleen is its enlargement, and this produces marked distress according to its size, the number and character of adhesions, and the interference with the functions of other organs. The sense of intolerable dragging when the tumor is large often demands removal, provided other conditions permit it. This enlargement is usually due to paludal, tubercular, or idiopathic causes, malignant disease, wandering of the organ, axial rotation, echinococcus and other cysts, chronic congestion, amyloid disease, and injuries, such as rupture. Perhaps the most frequent cause of enlargement of the organ—leucocythemia—is the one condition in which the operation of extirpation should almost never be performed. More than one hundred cases of splenectomy for leucocythemia have been reported, and in but three of them was it successful. Such results should be sufficient to put this indication for the operation practically beyond dispute. Some writers, however, believe the operation is justifiable when the relative proportion of red to white blood-cells is not lower than fifty to one. In some cases perhaps this proportion may not be markedly dangerous; but we must remember the spleen does reach a tremendous size in leucocythemia, often weighing from twenty to fifty pounds, and the adhesions may be formidable. In such cases certainly the fifty-to-one rule would be a very dangerous one, were it not that in these very marked cases of enlargement the proportion is usually less than the one mentioned. It shows, however, the necessity of knowing the blood conditions. Nor should the proportion of the red and white blood-cells be all we should attempt to learn in examination of the blood. It is well known that different varieties of the leucocytes have different functions, and that the relative numbers of this species of them vary very markedly in different specimens of blood, whether taken from the same or different patients. This is certainly an element of great importance. Aside from this, it is of the greatest moment to know the hemoglobin percentage of the blood of a patient suffering from a leucocythemic spleen. If this be not far below the normal, as shown by the present inaccurate instruments, the prognosis of the surgeon is less grave. A blood count of 4,000,000 red and 6,000 to 9,000 white blood-cells and a hemoglobin percentage above 70 per cent. would ordinarily be considered a safe blood condition for splenectomy. But should the red cells be 2,500,000 and the white ones increased to above 9,000, and perhaps the hemoglobin percentages be below 70, I would conclude splenectomy, under the most favorbly conditions otherwise, would be absolutely interdicted, unless death without operation seemed unavoidable. It will be noticed this blood-cell proportion is far above fifty to one. There has often been noticed a paludal intoxication accompanying enlargement of the spleen in people exposed to this germ that can only be removed when the spleen has been removed. This seemed to be so certain to Jonnesco and Vanverts that they have strongly recommended splenectomy in marked cases of malarial intoxication that did not yield to ordinary treatment. The effect of splenectomy on the conditions of the blood is often markedly emphasized in malarial hypertrophy. In wandering spleen there is great danger of sudden torsion of the pedicle, with very severe and dangerous results or adhesions to other organs, causing marked difficulties and grave dangers.

Splenectomy is not an operation to be lightly entered upon with scarcely any knowledge of the conditions demanding it and of those absolutely contraindicating it. It is fully as important to know when not to do the operation as when to intervene surgically.

As previously mentioned in the enlarged leucocythemic spleen, the conditions aside from those of the blood must be very good indeed to warrant entering on the procedure. If the organ be very large, the result will be less satisfactory, and if the adhesions be severe, the result is still more greatly jeopardized. Vanverts found in thirty-nine cases of enlargement with marked adhesions twenty-eight died, while in thirty-five without adhesions only two died. Profound cachexia is a very dangerous complication. Extreme enlargement of the tumor, especially if it be solid, is a signal disadvantage to the operation and to the patient, as it prevents easy manipulation of the pedicle in operation and tends to greatly add to the shock by the traction and extra manipulation during its removal. Of course, the general health of the patient will influence the result. If the patient be one that has always been unhealthy, the result will not be so good.

The history of my third case is as follows:

S. G., white, thirty-nine years of age, married, multipara, entered Columbia Hospital October 24, 1899. All her labors were normal; her youngest child is five years of age, and she has had no abortions. Her menses ceased in 1897. Her children had chills and fever at irregular intervals during the summer of 1894, and she, while having no chills, was ill with malaria at the same time and lived in that portion of the city near the eastern branch of the Potomac River. She first noticed a tumor in the left side of the abdomen in 1897, and since then has been unable to perform her usual household duties. Just before entering the hospital, a small ulcer appeared on the inner side of the interior surface of the left leg. This increased in size shortly after admission, and two small ones soon appeared near it. She was pale but in moderate flesh. She was immediately put in bed, as the pulse was 116 and her temperature above normal. A large mass was found in the abdomen extending from the diaphragm on the left side of the pelvic cavity, where it was easily felt by vagino-digital examinations. Two notches were found in the anterior margin of the mass and an enlarged spleen was diagnosed. During the first week in the hospital the pulse was high and the temperature ranged from 100° to 101° F. A blood examination, made on the 27th, showed red blood-cells, 4,600,000, and hemoglobin 90 per cent. Under active general and local treatment the ulcers were not healed until about December 18, on which day she was allowed to sit out of bed. In a few hours the healed surfaces became black and showed a tendency to slough. The left foot and right leg took on a marked petechial appearance which covered the rest of the body in a milder form. She was put in bed again. During her eight weeks in bed her general condition had not improved and the spleen had apparently enlarged. It was believed her condition, though unsatisfactory, could not be improved for operation. Blood examination, December 14, 1899, showed no malarial parasites; red blood-cells, 3,500,000, and varied greatly in size. One or two megalocytes and an occasional myelocyte; leucocytes approximately normal in number, the mononuclears being slightly increased. Several times examinations for malarial parasites were made with negative results. From the 12th to the 24th of November the temperature was again nearly the same as during her first week in the hospital, and the pulse ranged from 84 to 104. After that time the chart shows normal pulse and temperature. Operation was done December 21, 1899. The left kidney was found to be about twice its normal size, quite movable, and to the inner side of the hilum of the spleen. It was not disturbed. A number of urinalyses had shown no abnormality. The spleen, extending from the diaphragm to the pelvic cavity and resting on the uterus, had five notches in its anterior border. Four large arteries and a number of smaller ones was encountered in ligating the pedicle. Two of them, close together, were each about the size of the femoral, and the two others, an inch apart, were but slightly smaller. Each was ligated separately with strong silk, fearing to trust catgut for this work. The arteries were each first clamped in two places near the spleen and divided with scissors between these clamps. Ligatures were then placed on the proximal side of them, and lastly, the veins were similarly treated. Slight hemorrhage occurred from a large blood-vessel stump on the spleen side, but, as all the arteries had been previously secured, no harm resulted. It amounted only to a partial drainage of the spleen. Owing to the size and position of the enlarged organ, it was found impossible to first ligate and divide the gastro-splenic ligament as had been planned. To a considerable part of the splenic surface were adhered intestine, omentum, diaphragm, and parietal peritoneum. Two quarts of normal salt solution had been thrown under the breasts at the beginning of the operation, and the same amount was left in the peritoneal cavity as the pulse had now risen to 136. That evening her pulse was 114 and her temperature 97.4° F.; her temperature remained at about 99° until the evening of the 25th, when it went up to 101°, the highest point noted previously to the 18th of January, the last day it was registered. This rise lasted but three days. On this date the blood count showed red blood-cells, 4,192,000 and 8,000 white blood-cells. January 4, 1900, a blood examination was made by Dr. Carroll, who found no malarial parasites and nothing of moment. January 12 she was allowed to sit out of bed, and was discharged from the hospital thirteen days later. The leg ulcers had nearly healed and her appearance was much better.

During the first three days' post-operation she slept but little, and then seemed drowsy, sleeping most of the time and requiring to be awakened for nourishment. On the 29th she became restless and slightly delirious. From this time to January 7th, she frequently cried from no apparent cause, accused herself of having at some former time committed some unpardonable sin, and refused to be comforted. This condition became better, and believing her being in a room alone had a deleterious effect, she was put in the ward with other patients, and was almost immediately free from her vagaries.

On January 23 her weight was 115 pounds, and on February 5 she visited the hospital, and was again weighed and found to have gained twelve pounds. She had markedly improved in appearance, and was bright, cheerful and energetic in her actions. Her cheeks were rounded out and rosy. Her leg ulcers were healed. On February 8 Dr. Carroll found her hemoglobin percentage was 74 and other blood conditions normal.

REPORT OF DR. JAMES CARROLL, HOSPITAL PATHOLOGIST.

*Macroscopic.*—The organ presents the normal tongue shape, with a flattened, slightly concave inner surface, a distinctly convex outer surface, and somewhat tapering margins. It measures eleven and one-quarter inches at the smaller end, and a corresponding thickness of two and three-quarter inches and one and three-quarter inches at the ends respectively. The upper margin is convex and smooth, with the exception of a large fissure near the head. The lower margin is concave and deeply notched. The margins are everywhere thick and rounded. The color is dark red, apparently from congestion, and the consistency is rather softer than normal. The capsule is tense and smooth and stippled with indistinct grayish points, which represent the enlarged follicles beneath. The weight of the organ in the fresh state is four pounds and four ounces.

*Microscopic.*—The section reveals only a chronic passive congestion with hypertrophy of the pulp and follicles. There is no deposit of pigment and the tuberculæ are not involved. Some of the veins contain large numbers of leucocytes, but in others the number appears to be about normal. The conditions found may be briefly described as a congestive hypertrophy.

No argument is needed to prove the advisability of splenectomy in a case such as this.

The operation has now a mortality rate of about 13 per cent., and this would be too high were all severe cases of leucocythemia and malignant disease excluded. Severe adhesions and marked hypertrophy of the organ are necessarily potent factors in making a mortality rate for splenectomy. Hemorrhage and shock are the principal immediate causes of death. The blood-vessels of the enlarged spleen are often found to be tremendously large, and necessarily the blood loss is excessive in a very short time from even one of them should it be permitted at all. It is therefore strongly advisable to proceed with the utmost caution in severing and securing these vessels. If forceps are applied before ligation, as was done in this case, the operator should know they are reliable and that they will not unclamp or slip off. Then, too, the vessels should be ligated singly and well back from the cut ends on the central side, with the ligatures always passing through the tissue as it passes around the vessel. It should pass through between vessels and never through any of them. This is exceedingly important. For this purpose silk is more reliable than other material because it is most easily knotted without loosening. The arteries should be the first to be secured, as the organ is easily distended, and more blood is taken from the circulation if they are allowed to continue pumping blood into the spleen after the veins are tied or clamped. The gastro-splenic ligament should be first clamped and separated, as it permits the spleen to be lifted up and affords better access to the pedicle. If the hypertrophy is marked, this preliminary measure will be ofttimes impossible. Under such conditions the first vessels coming to view will have to be the first to be secured, and followed by the next coming into view, and this plan continued until all have been secured and separated. Removal of the tumor facilitates application of the ligatures.

Shock is produced by separation of many or dense adhesions, by manipulation of the organ during the process of removal, and by removal from the under side of the diaphragm of the pressure to which the lungs and heart, as well as the abdominal viscera, have become accustomed. If the tumor be large this is of great moment. This may be overcome by leaving a large amount of normal salt solution in the abdominal cavity when the abdomen is closed, or by throwing it into the colon.

In every case in which it is possible operation should be preceded by a careful study of the history of the case and careful blood analyses made. In my former paper, already mentioned, I took occasion to suggest the changes subsequent to operation, varied according to the relative normal condition of the organ removed. It is well known these storms of fever, high pulse-rate, and other manifestations of marked trophic and psychologic disturbances are often furious in the adult, and in the child or young animal practically absent. In the case of the much-altered spleen, such changes in the vicarious functionating economy have begun long before operation, and thus leave less to be done in that direction after splenectomy. It is probably for that reason that extirpation is not followed by the critical conditions incident to the sudden taxing of the compensating organs following removal of the normal spleen for rupture, torsion of the pedicle, and other conditions.

Splenectomy for malignant disease has not yet been satisfactory. This is due to the disease spreading from the spleen very early, and therefore rapidly appearing in other structures only to cause an early death. For echinococcus and other cysts the operation is very successful, as it has been for rupture of the organ. During the past two years twenty cases have been operated for rupture, and but two of them died. One of these fatal ones was severely mangled, and had a rupture of the liver as well as of the spleen.

Splenopexy has been recommended and practised abroad for movable or wandering spleen when the organ was less than three times its normal size. The principle of the operation was to form an extraperitoneal pocket by slitting the peritoneum, placing the spleen behind it after separating it sufficiently, and then suturing the flaps over it. It need not be of necessity in the normal region of the spleen. I have had no experience whatever with this operation, and for that reason am not in a position to approve or condemn it. However, my impressions are that it is an unwise procedure. There is danger of sloughing of the flaps after separation, and of marked dragging and consequent injury from such suspension of a mass of tissue weighing over twenty-one ounces.

